# Hypermagnesemia in a psychiatric hospital

**DOI:** 10.1002/pcn5.70155

**Published:** 2025-07-16

**Authors:** Ryo Takenaka, Kayo Emori, Hirotsugu Kawashima

**Affiliations:** ^1^ Department of Psychiatry Toyooka Public Hospital Toyooka Japan; ^2^ Department of Pharmacy Ainohanazono Hospital Ibaraki City Japan; ^3^ Department of Neuropsychiatry Kyoto University Graduate School of Medicine Kyoto Japan

**Keywords:** anticholinergic drug, antipsychotic drug, constipation, hypermagnesemia, magnesium oxide

## Abstract

**Aim:**

An association between constipation and psychiatric disorders has been suggested. While magnesium oxide (MgO) is frequently administered in Japan to treat constipation, there is a risk of developing hypermagnesemia. We aimed to investigate the characteristics of hypermagnesemia in patients admitted to psychiatric hospitals.

**Methods:**

Among all patients admitted to Ainohanazono Hospital between January 2020 and December 2023 who had undergone ≥1 blood test, we retrospectively investigated the risk of developing hypermagnesemia in those with or without MgO, patient characteristics associated with this risk, and characteristics that determined the MgO dosage.

**Results:**

Of 1120 patients, 532 (48%) were prescribed MgO. The risk of developing hypermagnesemia was 2.76 times higher for patients prescribed MgO than for those not prescribed MgO. Patient characteristics associated with the risk of developing hypermagnesemia were daily MgO dose (hazards ratio [HR] 2.66), serum creatinine level (HR 1.75), and female biological sex (HR 1.44). Regarding patient characteristics that determined the prescribed MgO dosage, concomitantly administered antipsychotic and anticholinergic drugs were associated with an increase in the amount prescribed (odds ratio [OR] 1.12 and OR 1.06, respectively). However, long‐term treatment duration (OR 0.91) and blood urea nitrogen levels (OR 0.95) were associated with a decrease in the dosage.

**Conclusion:**

We clarified the risk of MgO‐related hypermagnesemia, particularly for patients undergoing long‐term (>1 year) MgO administration. Antipsychotic and anticholinergic drug dosages slightly affected the increase in the MgO dosage. Regular blood sampling is needed to prevent hypermagnesemia, with a switch to a non‐MgO laxative when renal function is impaired.

## INTRODUCTION

The prevalence of chronic constipation is high among patients with psychiatric disorders, with prevalence rates being particularly high among older adult patients with psychiatric disorders.^1^ An association between psychiatric illness and chronic constipation has also been reported.^2^ In addition to daily lifestyle factors such as reduced physical activity and food and fluid intake, an association between constipation and antipsychotic and anticholinergic drug therapies has been reported.^3^ Moreover, in Japan, where the average inpatient age is reported to be increasing, constipation management is becoming increasingly important in psychiatric care.^4^

In Japan and Southeast Asia, magnesium oxide (MgO) is both recommended and widely used as a laxative to treat chronic constipation.^5^, ^6^, ^7^ However, among older adults and patients with renal failure, serum magnesium (Mg) levels are known to increase, and serious adverse effects^8^, ^9^ or death^10^ owing to hypermagnesemia have been reported. Furthermore, severe hypermagnesemia has been reported in patients with psychiatric disorders without apparent renal dysfunction.^11^ It has been proposed that impaired bowel motility causes colonic distention, which facilitates magnesium absorption and thereby exacerbates hypermagnesemia, even with a relatively small MgO dosage,^10^, ^11^ therefore regular monitoring is recommended for patients prescribed MgO to prevent MgO‐related hypermagnesemia.^12^, ^13^

Some studies have identified factors that cause hypermagnesemia owing to oral MgO preparations, including one report involving older adult outpatients,^14^ two studies reporting on multiple risk factors for inpatients at general hospitals,^15^, ^16^ and one study involving inpatients at psychiatric hospitals that investigated the association between factors such as renal function, sex, and the amount of oral MgO in a single test.^17^ However, in addition to oral MgO dosages, treatment duration may also affect the development of hypermagnesemia.

In Japan, despite many patients requiring long‐term hospitalization in psychiatric hospitals, no studies have undertaken comparisons between the incidence of hypermagnesemia and treatment duration. Moreover, no studies have examined risk factors for developing hypermagnesemia owing to long‐term MgO use in a hospital environment. We therefore aimed to retrospectively examine multiple risk factors for hypermagnesemia among psychiatric hospital inpatients who had been prescribed MgO, with consideration to the duration of treatment. We also retrospectively examined the factors affecting MgO dosage, including anticholinergic and antipsychotic drug administration.

## METHODS

### Participants

All patients who had been admitted to Ainohanazono Hospital between January 2020 and December 2023 and had undergone ≥1 blood test were included in the study. We excluded patients with missing values for the measured variables and those diagnosed with hypermagnesemia, based on the first blood test after admission.

### Measurements

The variables investigated included age, sex, serum creatinine level, blood urea nitrogen (BUN) level, daily MgO dose, daily antipsychotic medication dose, and daily anticholinergic medication dose. Chlorpromazine and biperiden equivalents were calculated using established conversion tables.^18^

### Outcomes

The primary outcome was the hazard ratio (HR) for the onset of hypermagnesemia. Secondary outcomes included patient characteristics related to the onset of hypermagnesemia and those that determined the MgO dosage. Hypermagnesemia was defined as a serum Mg level >2.5 mg/dL, in accordance with the Common Terminology Criteria for Adverse Events version 5.0.^19^

### Statistical analyses

Patients selected in accordance with our inclusion and exclusion criteria were divided into two groups, namely, those who were regularly prescribed MgO (a prescription group) and those who were not (a control group).

Descriptive analyses were performed to describe the study population's characteristics and outcomes. Continuous variablesweare summarized as means and standard deviations (SDs) based on their distribution, and categorical variables were expressed as absolute and relative frequencies. Hypermagnesemia occurrence and severity were assessed using CTCAE v5.0 and classified into four grades (ranging from Grade 0, values within a normal range, to Grade 3, values showing the highest severity). A Fisher's exact test was used to compare hypermagnesemia occurrence between the two groups.

Using the Kaplan–Meier method, we first analyzed the association between the first occurrence of hypermagnesemia and the use of MgO, and then fitted a Cox proportional hazards regression model. For the prescription group, the first prescription date during the observation period was deemed the starting date, whereas the starting date for the control group was either the date of hospitalization or January 1, 2020, which was deemed the start of the observation period. The analysis period was calculated from the date of the first onset of hypermagnesemia as an event, or from the last examination date during the observation period as the end date. The first hospitalization during the observation period was used for patients with a history of multiple hospitalizations. In the Kaplan–Meier analysis, the cumulative incidence was first calculated. The cumulative incidence of hypermagnesemia in the MgO prescription and control groups was evaluated using Gray's test. For the Cox proportional hazards regression model, a univariate Cox proportional hazards model was first applied. The risk of hypermagnesemia development was also analyzed for each prescription period. The HR for the onset of hypermagnesemia was analyzed by dividing the observation period into <6 months, 6–12 months, and >12 months. Multivariate Cox regression analysis was then performed. A significance level of 0.10 was set as the criterion for excluding variables from the final multivariate Cox model, followed by a stepwise backward selection procedure. In this analysis, the presence or absence of hypermagnesemia was used as the dependent variable, and the following independent variables were included: age, sex, serum creatinine level, BUN level, daily MgO dose, daily antipsychotic drug dose, and daily anticholinergic drug dose at the time of event occurrence or censoring. Wald's test was used to evaluate whether each variable had a statistically significant effect on survival time. The results of the covariates included in the final model were reported as HRs and 95% confidence intervals (CIs). In addition, Schoenfeld's general test was used in the multivariate Cox proportional hazards regression analysis to confirm that there were no issues with the fit.^20^

A generalized linear model was then used to analyze factors affecting the daily MgO dose. A significance level of 0.10 was set as the criterion for excluding variables, which were identified using the stepwise backward selection procedure. Multivariate regression analysis was performed with the MgO daily dose as the dependent variable. Age, sex, serum creatinine level, BUN level, daily doses of concomitantly administered antipsychotic and anticholinergic drugs, and the duration of MgO administration were the independent variables. Wald's test was used to evaluate whether each variable had a statistically significant effect on the amount of MgO prescribed as an explanatory variable. The results included in the final model are expressed as odds ratios (ORs) and 95% CIs. Statistical analyses were performed using R (v.4.2.0) and EZR (v.1.64)^21^ software. Statistical significance was set at P < 0.05.

## RESULTS

### Patient background

Of 1242 patients initially included in this study, 13 were excluded because of missing data in relation to age, BUN levels, blood Mg levels, or a primary diagnosis at the time of admission. A further 109 patients with elevated blood Mg levels in the first blood test after admission were also excluded. Finally, 1120 patients were included in this retrospective analysis.

The demographic and clinical characteristics of the 1120 study patients are shown in Table 1. The average patient age was 64.1 years, the average BUN level was 13.3 mg/dL, the average serum creatinine level was 0.71 mg/dL, and the average blood Mg level was 2.2 mg/dL. No abnormalities were observed in the average test data. Regarding biological sex, 511 (46%) patients were women, 111 (10%) patients were undergoing anticholinergic drug therapy, and 620 (55%) patients were undergoing antipsychotic therapy. The main diagnosis at the time of admission was categorized in accordance with ICD‐10 codes, as follows: organic disorders, including dementia, substance use disorders, schizophrenia and delusional disorders, mood disorders, and others. Our findings are summarized in Table 2. The largest diagnostic group was schizophrenia‐related (*n* = 600, 53.6%), followed by those with organic disorders, including dementia (*n* = 253, 22.6%), mood disorders (*n* = 135, 12.1%), and substance use disorders (*n* = 59, 5.3%). At the start of the study in January 2020, 243 patients were long‐term inpatients with a hospitalization period of >12 months.

**Table 1:** Demographic and clinical characteristics of 1120 patients at the first blood test.

	Prescription (*n* = 527)	Control (*n* = 593)	*p* value
Age (years)	65.06 ± 13.87	63.27 ± 17.56	0.061
Blood Mg level (mg/dL)	2.28 ± 0.2	2.14 ± 0.21	<0.01
Serum creatinine level (mg/dL)	0.67 ± 0.23	0.75 ± 0.37	<0.01
BUN level (mg/dL)	12.61 ± 5.34	13.84 ± 7.74	<0.01
Sex, female: *n* (%)	252 (47.8%)	259 (43.7%)	0.184
Taking anticholinergic drugs *n* (%)	82 (15.6%)	29 (4.9%)	<0.01
Taking antipsychotic drugs *n* (%)	429 (81.4%)	191 (32.2%)	<0.01

*Note*: Continuous variables are presented as mean ± standard deviation; binary variables are presented as absolute numbers (percentages). A Student's *t*‐test was used for continuous variables; a chi‐square test was used for binary variables.

Abbreviations: BUN, blood urea nitrogen; *n*, number; Mg, magnesium.

**Table 2:** Psychiatry diagnosis at the time of admission, based on ICD‐10 codes.

	Prescription (*n* = 527)	Control (*n* = 593)	p value
Schizophrenia and delusional disorders	288	312	<0.01
Organic disorders including dementia	142	111	0.28
Mood disorders	82	53	0.065
Substance use disorders	35	24	0.382
Others	46	27	0.097

*Note*: A chi‐square test was used to evaluate the P‐values.

The average MgO dose was 1000 mg (SD: 480 mg). Hypermagnesemia occurrence and severity, which were assessed using CTCAE v5.0, are summarized in Table 3. Compared with the control group, a significantly higher proportion of patients with Grade 1 or Grade 3 hypermagnesemia was observed in the prescription group (Fisher's exact test, P < 0.01).

**Table 3:** Occurrence and severity of hypermagnesemia based on CTCAE v5.0.

Grade	Prescription (*n* = 527)	Control (*n* = 593)	P‐value
Grade 0	326	521	<0.01
Grade 1	194	71	
Grade 3	7	1	

*Note*: Fisher's exact test was used to evaluate the P‐values.

### Analysis using the Kaplan–Meier method

**Figure 1:**
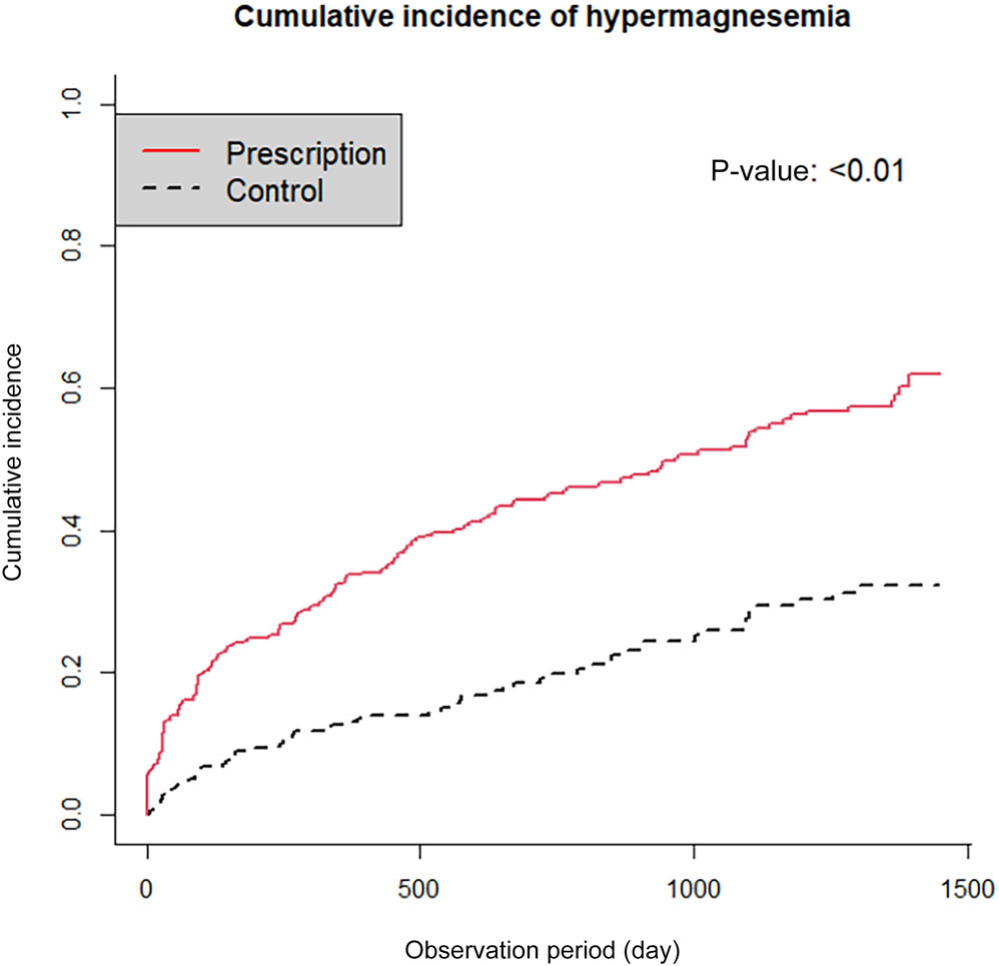
Inverse Kaplan–Meier curve for the cumulative incidence of hypermagnesemia with or without oral MgO administration.

Of 1120 patients with normal blood Mg levels at the initial blood test, 532 (48%) received prescriptions for MgO during the observation period (prescription group) and 588 (52%) did not (control group). During 447,412 person‐days of risk analysis within the observation period, 273 cases of hypermagnesemia were identified during the observation period. When stratified according to whether MgO was prescribed, 201 of 532 (38%) patients in the MgO group and 72 of 596 (12%) patients in the control group developed hypermagnesemia. The incidence rates per 500 patient‐days were 0.390 in the prescription group and 0.141 in the control group; per 1000 patient‐days, the incidence rates were 0.507 in the prescription group and 0.245 in the control group. This difference was assessed using Gray's test, with a statistical value of 59.9, and P < 0.01 was considered significant. Figure 1 shows the cumulative incidence of hypermagnesemia during the observation period in patients with and without a prescription of MgO, using an inverse Kaplan–Meier curve.

### Analysis using a Cox proportional hazards regression model

Univariate Cox proportional hazard regression analysis showed that the prescription group had an increased risk of hypermagnesemia compared with the control group (HR 2.76, 95% CI 2.11–3.61, P < 0.001). The HRs for the development of hypermagnesemia when the observation period was divided into <6 months, 6–12 months, and >12 months were as follows: HR 3.25 (95% CI 2.22–4.75, P < 0.01), HR 2.66 (95% CI 1.25–5.66, P = 0.01), and HR 2.17 (95% CI 1.39–3.40, P < 0.01), respectively. For all observation periods, the findings indicated that the risk of hypermagnesemia was higher in the prescription group. Table 4 shows the HRs of univariate the Cox proportional hazards regression analysis when the observation period was divided into three groups.

**Table 4:** HRs for the development of hypermagnesemia based on univariate Cox proportional hazards analysis after having divided the observation period into <6 months, 6–12 months, and >12 months.

Period (months)	Sample size (*n*)	Events	HR	Lower 95% CI	Upper 95% CI	P‐value
0–6	1,120	147	3.25	2.22	4.75	<0.01
6–12	524	36	2.66	1.25	5.66	0.011
>12	391	90	2.17	1.39	3.40	<0.01

*Note*: A log‐rank test was used to evaluate the P‐values.

*Note*: CI, confidence interval; HR(s), hazard ratio(s); *n*, number.

**Figure 2:**
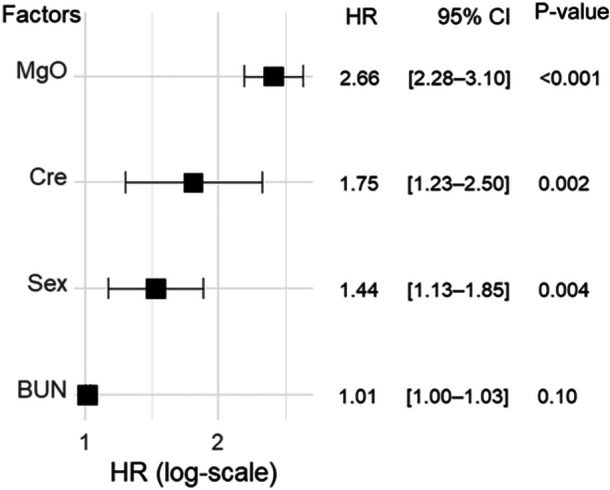
Forest plot of factors associated with hypermagnesemia development with or without oral MgO administration, using a multivariate Cox proportional hazards regression model. Wald's test was used to evaluate the P‐value. BUN, blood urea nitrogen; CI, confidence interval; Cre, creatinine, HR, hazard ratio; MgO, magnesium oxide.

Among the variables evaluated, sex, serum creatinine level, daily MgO dose, and the BUN level were included in the final model. In the final multivariate Cox proportional hazard regression analysis, the daily MgO dose (HR 2.66, 95% CI 2.28–3.10, P < 0.001), serum creatinine level (HR 1.75, 95% CI 1.23–2.50, P = 0.002), and female sex (HR 1.44, 95% CI 1.13–1.85, P = 0.004) were shown to be statistically significantly associated with the first occurrence of hypermagnesemia. Figure 2 shows a forest plot of the final Cox proportional hazards regression model, including variables that did not show a significant difference.

### Analysis using generalized linear models

**Figure 3:**
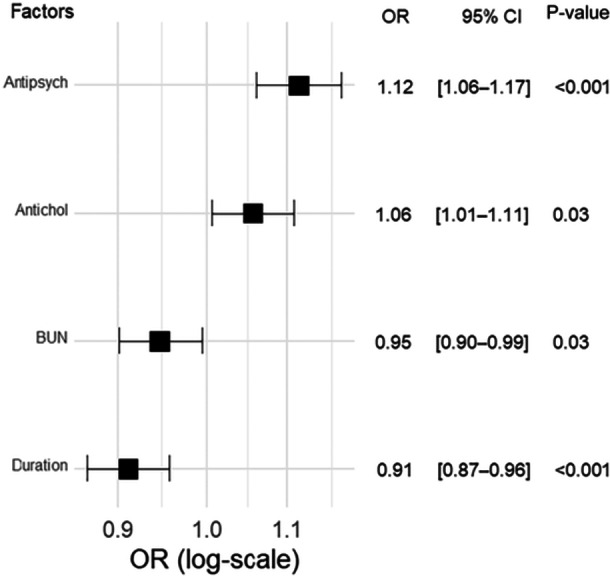
Forest plot of factors that predict the amount of MgO: a generalized linear model for 527 patients who received MgO prescriptions. Wald's test was used to evaluate the P‐value. Antichol, anticholinergic; Antipsych, antipsychotics; BUN, blood urea nitrogen; CI, confidence interval; OR, odds ratio.

Among the 1120 patients whose blood Mg levels were normal at the time of the initial measurement, we analyzed the factors that predicted the MgO prescription dosage using a generalized linear model for 527 patients who had received prescriptions for MgO. We then investigated the characteristics that were associated with an increase in the MgO dosage. Among the variables evaluated, the daily dose of concomitantly administered antipsychotic drugs, the daily dose of concomitantly administered anticholinergic drugs, the duration of oral administration, and BUN levels were not excluded and were incorporated into the final model. The generalized linear model showed that the daily dose of concomitantly used antipsychotic drugs (OR 1.12, 95% CI 1.06–1.17, P < 0.001) and the daily dose of anticholinergic drugs (OR 1.06, 95% CI 1.01–1.11, P = 0.03) were statistically significantly associated with an increase in the prescription MgO dosage. However, the duration of oral administration (OR 0.91, 95% CI 0.87–0.96, P < 0.001) and the BUN level (OR 0.95, 95% CI 0.90–0.99, P = 0.03) were statistically significantly associated with a decreased MgO dosage. Figure 3 presents a forest plot of the final generalized linear model.

## DISCUSSION

This cohort study is the first to analyze the effects of the long‐term oral MgO administration on the development of hypermagnesemia. Our findings indicated that oral MgO administration significantly increases the risk of hypermagnesemia in psychiatric inpatients admitted to psychiatric hospitals, and that there is a risk of developing hypermagnesemia even among patients with long‐term prescriptions that have been administered for >12 months. There was a 2.76 times greater risk of developing hypermagnesemia in patients taking oral MgO compared with those not taking MgO. Furthermore, multivariate COX proportional hazard analysis identified the following risk factors for the development of hypermagnesemia during the administration of MgO: daily dose of MgO preparations, serum creatinine levels, and female biological sex.

Our study findings are similar to those of Wakai et al.,^15^ who examined the risk factors for hypermagnesemia in hospitalized patients at a general hospital, in showing that reduced renal function and daily MgO doses were risk factors for hypermagnesemia development. These findings are consistent with previous reports^14^, ^16^ that have identified reduced renal function and high MgO dosage as risk factors for hypermagnesemia. Wakai et al.^15^ also identified age as a risk factor; however, it was not identified as a risk factor in this study, possibly because our study patients’ characteristics differed in that the average patient age in our study was 63.9 years, whereas Wakai et al. reported an average patient age of 42 years. As our entire patient cohort was older, we consider that differences owing to age were not a contributing factor in our analysis.

In Wakai et al.'s study, biological sex was also not recognized as a significant risk factor; however, in this study, it was shown to be significantly different. This is because previous studies used the estimated glomerular filtration rate (eGFR) to evaluate renal function. Given the eGFR differs depending on biological sex, it is possible that the elements of biological sex were confounded. In addition, previous studies have not evaluated the effects of taking MgO for longer periods (>12 months). A high risk of hypermagnesemia was observed in the early stages of treatment and for those under MgO administration for longer durations, even >12 months.

The results of our generalized linear regression analysis showed that the daily dose of antipsychotic drugs and the daily dose of anticholinergic drugs used in combination increased the MgO dosage, but the respective ORs were 1.12 and 1.06, therefore the effect on the amount prescribed was not considered significant. While the duration of oral administration and BUN levels decreased with the MgO dosage, the respective ORs were 0.91 and 0.95, therefore the effect on the dose prescribed was not significant.

Our study had some limitations. The participants were patients admitted to a psychiatric hospital, and the data were analyzed retrospectively. We used data from a specific region and medical institution, and the characteristics of the target population may differ from those of the general population with psychiatric disorders in other psychiatric hospitals, therefore caution is needed when generalizing these results to other regions or to different patient populations, such as outpatients, patients in psychiatric emergency wards, and patients with psychiatric disorders in general hospitals. There may be a delay between the actual onset of hypermagnesemia and its detection, which could have led to an underestimation of the time‐to‐event in the Kaplan–Meier analysis. Thus, the onset of hypermagnesemia in the prescription group may have occurred earlier than estimated. Furthermore, while we statistically adjusted for confounding factors, it was challenging to fully consider all potential confounding factors, and there is a possibility that other unadjusted factors may have affected the results. We were unable to adjust for certain potential covariates such as dietary intake, the use of medications affecting metabolism (e.g., a loop diuretic or activated vitamin D), and the severity of constipation. A decreased dietary intake may reduce the risk of developing hypermagnesemia, while severe constipation could increase that risk. In terms of medications, depending on the type and usage, for example, a loop diuretic may reduce the risk whereas activated vitamin D might increase the risk.

The study findings provide important real‐world data regarding the development of hypermagnesemia with MgO administration, particularly for patients undergoing long‐term administration of >12 months. Furthermore, the concomitant use of antipsychotic and anticholinergic drugs was associated with a modest increase in the prescribed amount of MgO. The collected data confirmed the need for regular blood monitoring to prevent the onset of hypermagnesemia and to switch to a laxative other than MgO when renal test results indicate a decline in renal function.

## AUTHOR CONTRIBUTIONS

**Kayo Emori**: Administrative, technical, or material support. **Hirotsugu Kawashima**: Study supervision; writing—review and editing. **Ryo Takenaka** is the guarantor of this work, had full access to all of the data in the study, takes responsibility for the integrity of the data and the accuracy of the data analysis, and wrote the manuscript. All authors approved the final version of the manuscript.

## CONFLICT OF INTEREST STATEMENT

No potential conflicts of interest relevant to this article were reported.

## ETHICS APPROVAL STATEMENT

This study protocol was approved by the Institutional Review Board at Ainohanazono Hospital (approval No.23‐006).

## PATIENT CONSENT STATEMENT

Based on the ethical guidelines, an opt‐out consent method was chosen.

## CLINICAL TRIAL REGISTRATION

Not applicable.

## Data Availability

The datasets generated during and/or analyzed during the current study are not publicly available as this was a single‐center study investigating patient medical records. However, data are available from the corresponding author on reasonable request.
